# Supplementary material for: Evolutionary and structural basis of SLAMF1 utilization in morbilliviruses—Implications for host range and cross-species transmission
Source: PLoS Pathog. 2025 Jun 10;21(6):e1012990. doi: 10.1371/journal.ppat.1012990 (PMC12180634; doi:10.1371/journal.ppat.1012990)
Supplement: S1 Table — IFIE and PIEDA energy components between 359th residue of MV-H and each residue of SLAM. (DOCX) [file ppat.1012990.s001.docx]

**S1 Table**

**IFIE and PIEDA energy components between 359^th^ residue of MHV and each residue of SLAM**

| MV-H  (WT) | SLAM | ES | EX | CT+mix | DI | IFIE |
| --- | --- | --- | --- | --- | --- | --- |
| Tyr539 | Glu71 | 2.9 | 0.0 | 0.0 | 0.0 | 2.9 |
| Tyr539 | Asn72 | -1.0 | 0.0 | 0.0 | -0.2 | -1.2 |
| Tyr539 | Ser73 | -0.4 | 0.0 | 0.0 | -0.1 | -0.6 |
| Tyr539 | Val74 | -0.5 | 5.1 | -2.5 | -5.6 | -3.6 |
| Tyr539 | Glu75 | 5.2 | 0.8 | 1.0 | -1.6 | 5.5 |
| Tyr539 | Asn76 | -1.3 | 0.0 | 0.0 | -0.1 | -1.4 |
| sum | | 4.9 | 5.9 | -1.5 | -7.5 | 1.7 |

| MV-H  (Y539D) | SLAM | ES | EX | CT+mix | DI | IFIE |
| --- | --- | --- | --- | --- | --- | --- |
| Asp539 | Glu71 | 46.2 | 0.0 | -0.2 | -0.3 | 45.8 |
| Asp539 | Asn72 | -10.3 | 0.8 | -2.4 | -2.2 | -14.1 |
| Asp539 | Ser73 | -22.5 | 0.2 | -0.8 | -1.0 | -24.1 |
| Asp539 | Val74 | 5.9 | 0.1 | -0.9 | -1.0 | 4.1 |
| Asp539 | Glu75 | 33.6 | 0.0 | 0.0 | -0.1 | 33.5 |
| Asp539 | Asn76 | -5.7 | 0.0 | 0.0 | 0.0 | -5.7 |
| sum | | 47.3 | 1.0 | -4.2 | -4.6 | 39.5 |
