# Supplementary material for: Evolutionary and structural basis of SLAMF1 utilization in morbilliviruses—Implications for host range and cross-species transmission
Source: PLoS Pathog. 2025 Jun 10;21(6):e1012990. doi: 10.1371/journal.ppat.1012990 (PMC12180634; doi:10.1371/journal.ppat.1012990)
Supplement: S2 Table — GenBank accession numbers for morbillivirus H/RBP and F genes. (DOCX) [file ppat.1012990.s002.docx]

**S2 Table**

**GenBank accession numbers for morbillivirus H/RBP and F genes.**

| Host | Strain | Gene | GenBank accession number |
| --- | --- | --- | --- |
| MV | IC-B | H | NC_001498.1 |
|  |  | F | NC_001498.1 |
| CDV | Ac96I | H | AB753775 |
|  |  | F | AB753775 |
| PPRV | Ghana/NK1/2010 | H | KJ466104 |
|  |  | F | KJ466104 |
| RPV | KabeteO | H | NC_006296 |
|  |  | F | NC_006296 |
| PDV | 982A | H | MT066171 |
|  |  | F | MT066172 |
| CeMV | muc | H | MT066173 |
|  |  | F | MT066174 |
| MBaMV | Not available | H | MW553715 |
|  |  | F | MW553715 |
