# Supplementary figures and images for: Evolutionary and structural basis of SLAMF1 utilization in morbilliviruses—Implications for host range and cross-species transmission
Source: PLoS Pathog. 2025 Jun 10;21(6):e1012990. doi: 10.1371/journal.ppat.1012990 (PMC12180634; doi:10.1371/journal.ppat.1012990)

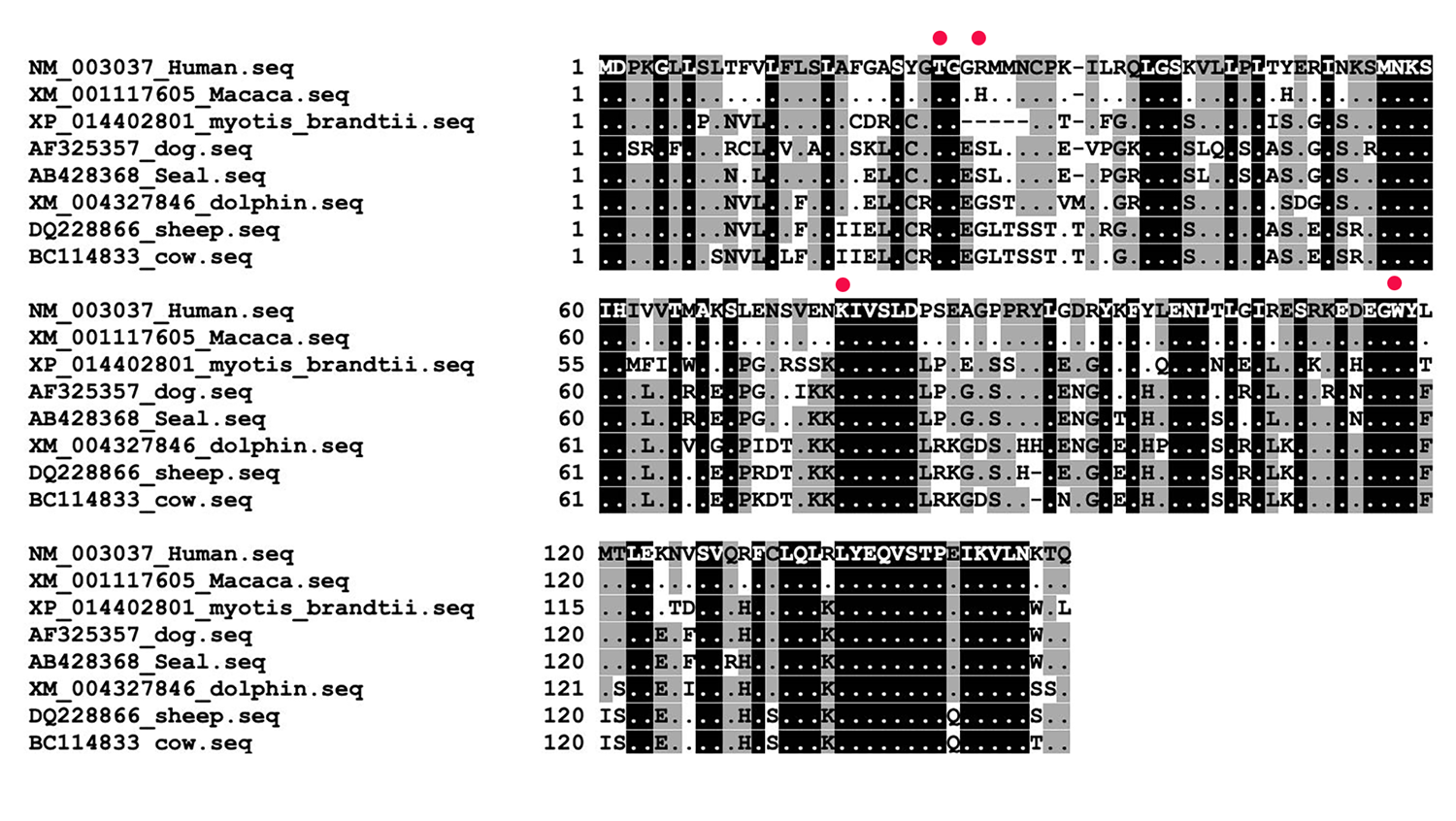

Supplement: S1 Fig — Amino acid sequences of SLAMs from various animals were obtained from GenBank and aligned using the ClustalW program in Genetyx-Mac software ver. 21. Residues identical to the top sequence are represented by dots, while mismatched residues are shown as their respective amino acid letters. Conserved residues across all sequences are highlighted in black, whereas residues conserved in more than half of the sequences are highlighted in gray. Deleted amino acid residues are indicated by hyphens. The amino acid positions shown to affect the H-SLAM interaction in this study are indicated by red dots. (TIF) [file ppat.1012990.s003.tif]

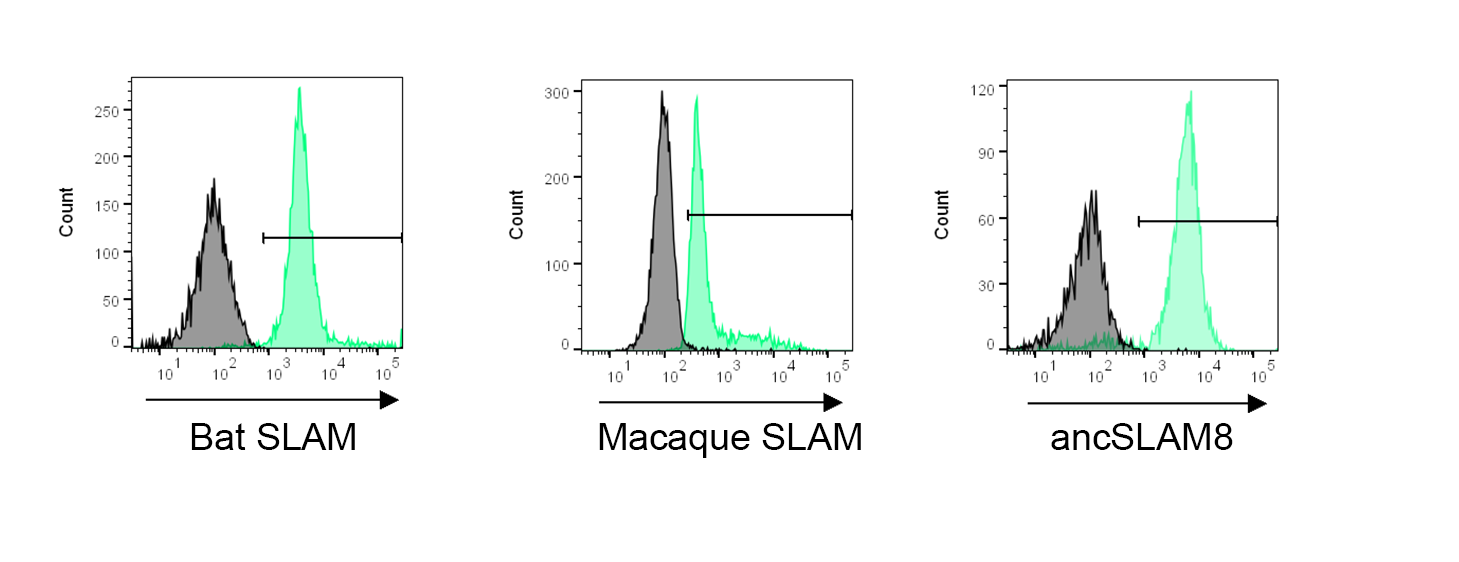

Supplement: S2 Fig — Vero.BatSLAMtag (filled green profile, left panel), Vero.ancSLAM8tag (filled green profile, right panel), and parental Vero cells (filled gray profile, left and right panels) were stained with a mouse anti-HA tag monoclonal antibody (clone 16B12, BioLegend), followed by Alexa Fluor 488-conjugated anti-mouse IgG staining. Vero/macSLAM-6 (filled green profile, center panel) and parental Vero cells (filled gray profile, center panel) were stained with a mouse anti-SLAM monoclonal antibody (clone IPO-3, Kamiya Biomedical), followed by Alexa Fluor 488-conjugated anti-mouse IgG staining. (TIF) [file ppat.1012990.s004.tif]

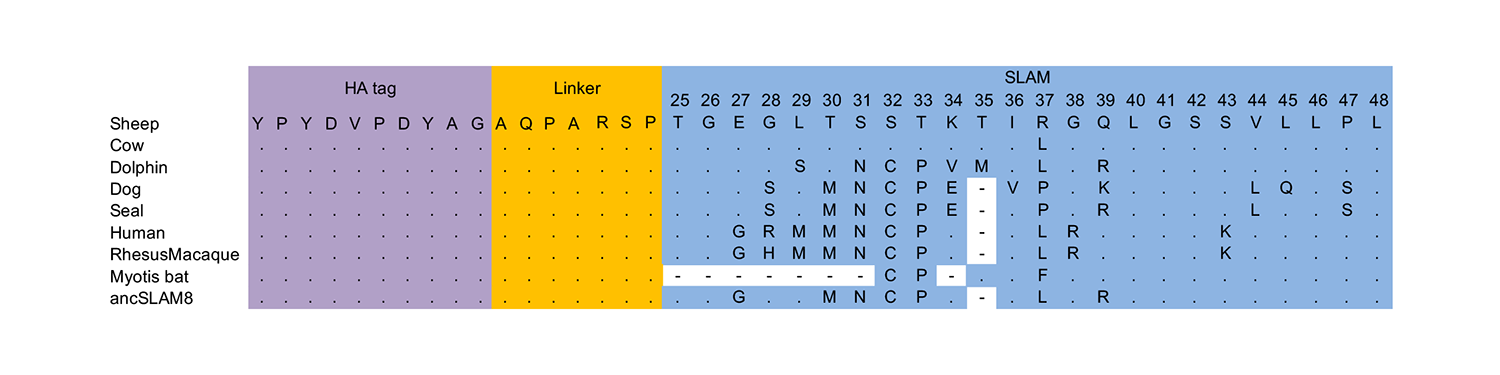

Supplement: S3 Fig — The HA tag sequence, SLAM sequence, and linker connecting the HA tag and SLAM sequence are highlighted in purple, blue, and orange, respectively. Residues identical to the top sequence are represented as dots, while mismatched residues are shown as their corresponding amino acid letters. Deleted amino acid residues are indicated by hyphens in the alignment analysis. Accession numbers: DQ228866.1 (sheep), BC114833.1 (cow), XM_004327846.1 (bottlenose dolphin), AF325357 (dog), AB428368 (spotted seal), NM_003037.4 (human), and XP_014402801.1 (riparian myotis bat). and XM_001117605.3 (macaque). (TIF) [file ppat.1012990.s005.tif]

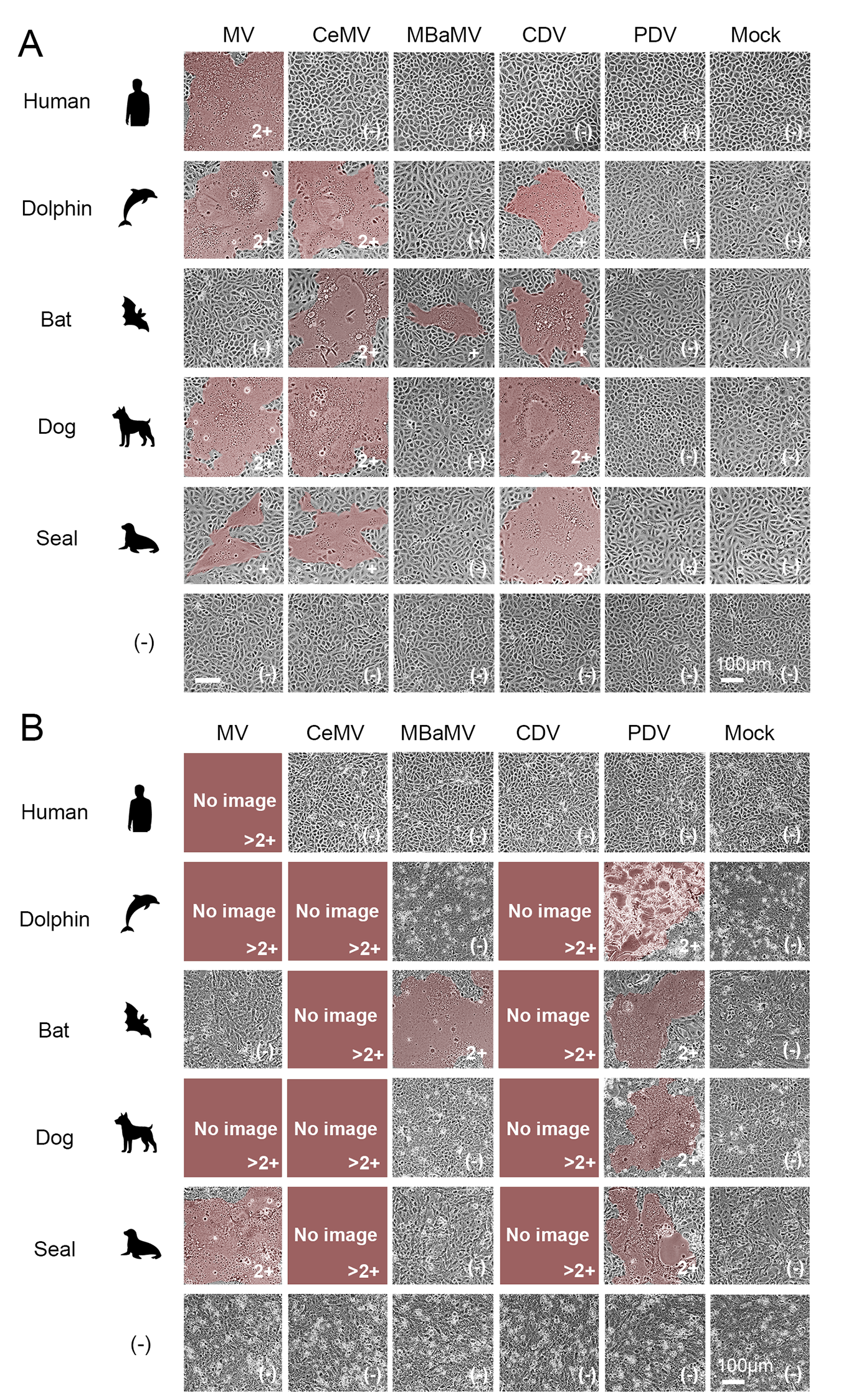

Supplement: S4 Fig — Vero cells stably expressing SLAM from different animal species (human, dolphin, bat, dog, and seal), along with parental Vero cells (denoted as ‘-’), were infected or mock-infected with measles virus (MV), cetacean morbillivirus (CeMV), myotis bat morbillivirus (MBaMV), canine distemper virus (CDV), or phocine distemper virus (PDV) at a multiplicity of infection (MOI) of 0.01. Cytopathic effects (CPEs) were evaluated at 24-hour intervals. (A) CPE observations at 24 hours post-infection (hpi). (B) CPE observations at 96 hours post-infection. CPE scoring: 2 + : Large syncytia observed throughout the field of view; + : Few small syncytia detected; (-): No syncytia detected; > 2 + : Majority of cells detached (no image shown). Syncytial areas are highlighted in brown. Animal silhouette images were generated using OpenAI’s image generation system (DALL·E) and are published under the terms of the Creative Commons Attribution 4.0 International License (CC BY 4.0). For terms of use, see https://openai.com/policies/terms-of-use. (TIF) [file ppat.1012990.s006.tif]

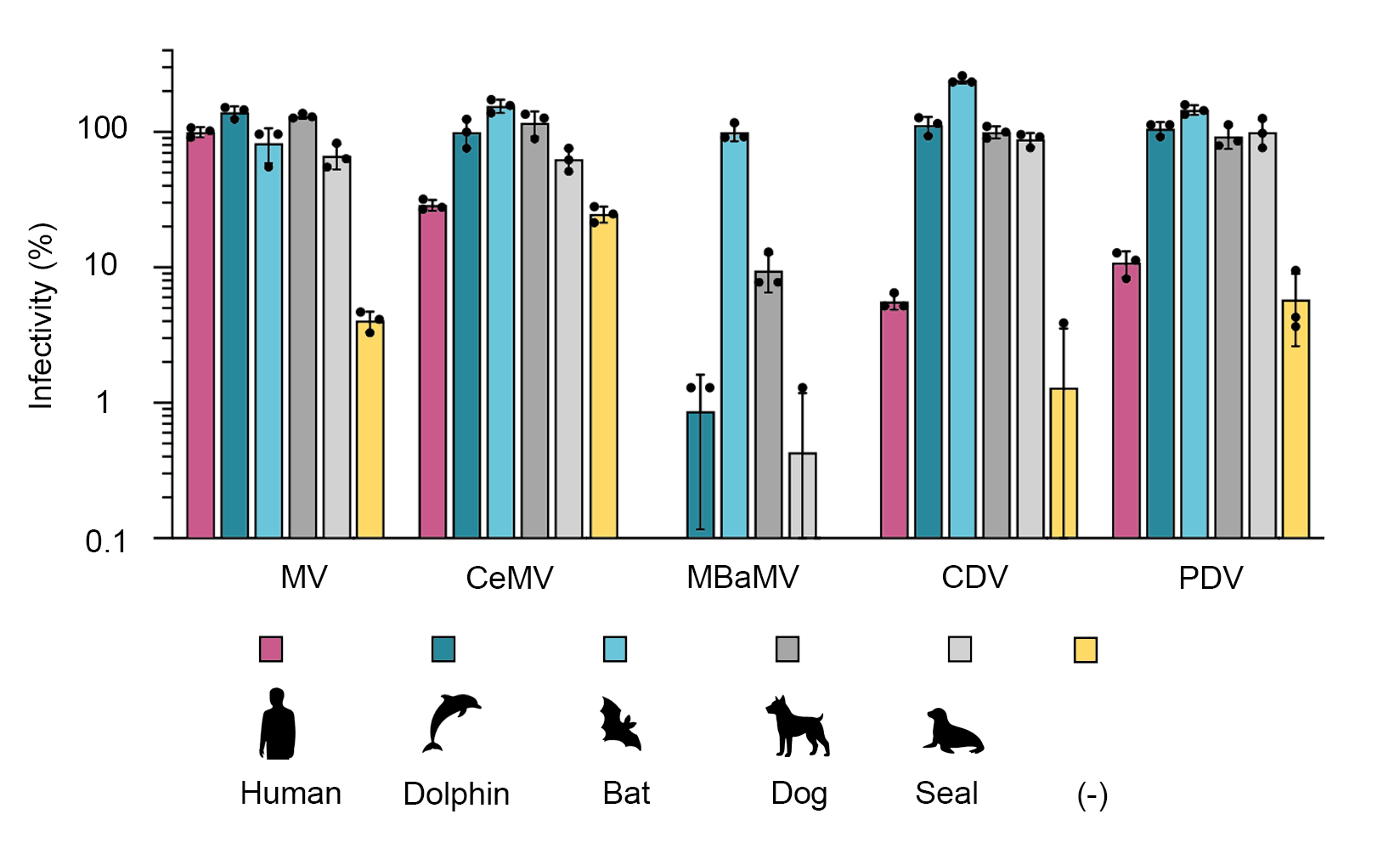

Supplement: S5 Fig — Vero cells stably expressing SLAM from different animal species (human, dolphin, bat, dog, and seal), along with parental Vero cells (denoted as ‘–’), were incubated with tenfold serial dilutions of virus samples (MV, CeMV, MBaMV, CDV, or PDV) for 1 hour. At 18 hours p.i., the cells were fixed with PBS containing 10% formalin, and permeabilized with 0.2% Triton X-100. Infected cells were detected using primary monoclonal antibodies against the MV N protein (clones A56 and E137), followed by Alexa Fluor 488-conjugated secondary antibodies. Fluorescent signals were visualized using a fluorescence microscope, and fluorescent infectious foci were counted. Animal silhouette images were generated using OpenAI’s image generation system (DALL·E) and are published under the terms of the Creative Commons Attribution 4.0 International License (CC BY 4.0). For terms of use, see https://openai.com/policies/terms-of-use. (TIF) [file ppat.1012990.s007.tif]

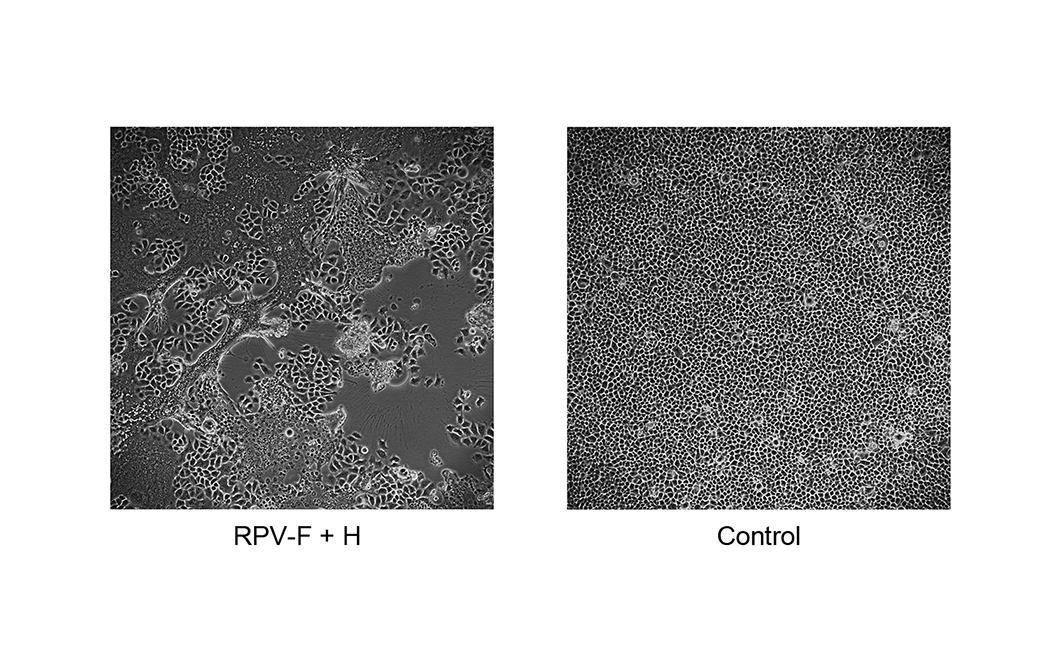

Supplement: S6 Fig — The RPV H and F proteins were expressed in HeLa cells using expression plasmids. As a control, the RPV F protein was also expressed alone in HeLa cells. One day post-transfection, the cells were observed under a microscope. (TIF) [file ppat.1012990.s008.tif]

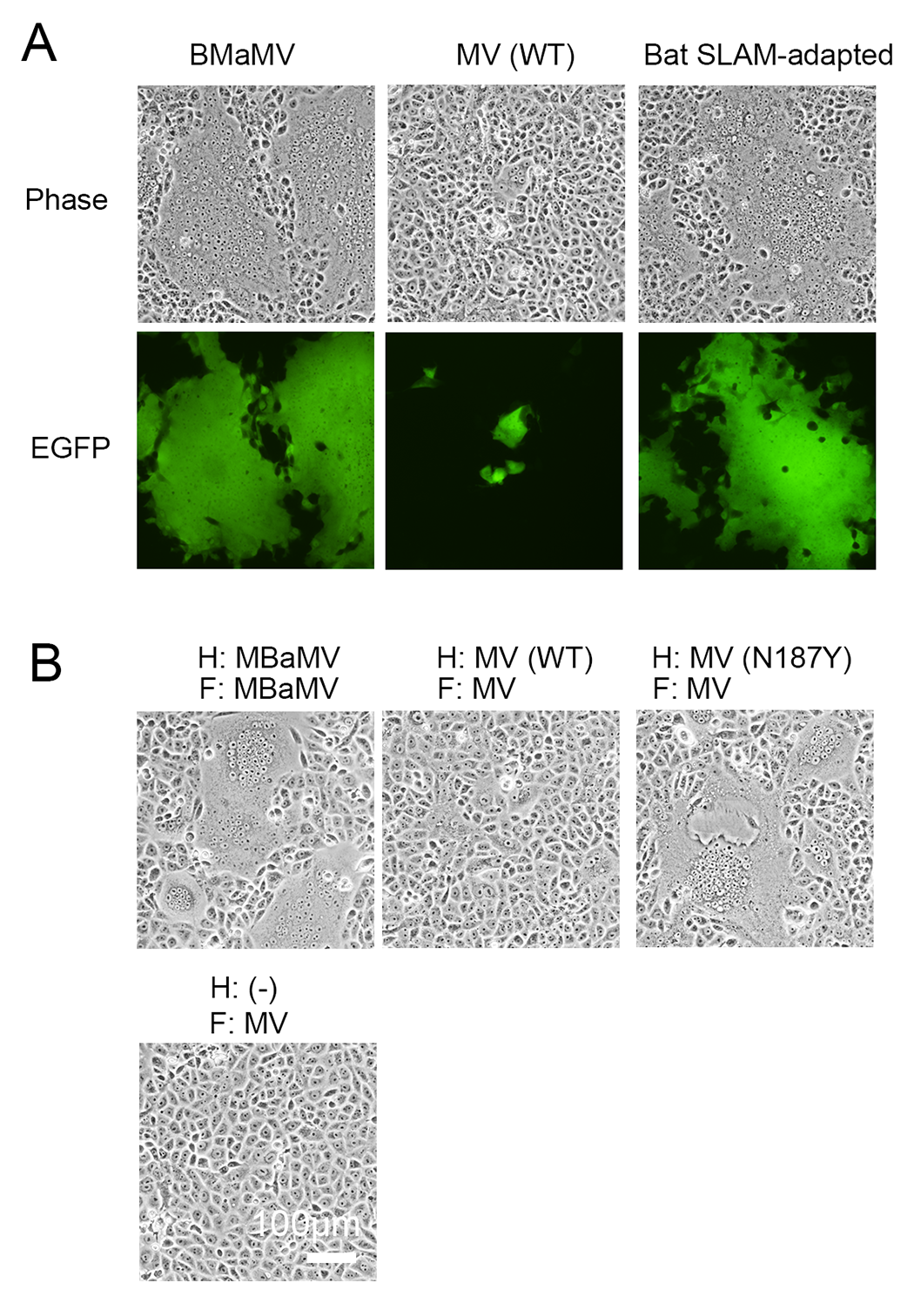

Supplement: S7 Fig — (A) Syncytium formation by EGFP-expressing MBaMV, wild-type MV, and bat SLAM-adapted MV. (B) Syncytium formation by the H protein and F protein-encoding plasmids. The H and F proteins of MBaMV or MV were expressed in Vero cells expressing bat SLAM with an authentic, unmodified N terminus using expression plasmids. The MV H protein carrying the N187Y mutation was also expressed in these cells. One day post-transfection, the cells were observed under a microscope. (TIF) [file ppat.1012990.s009.tif]

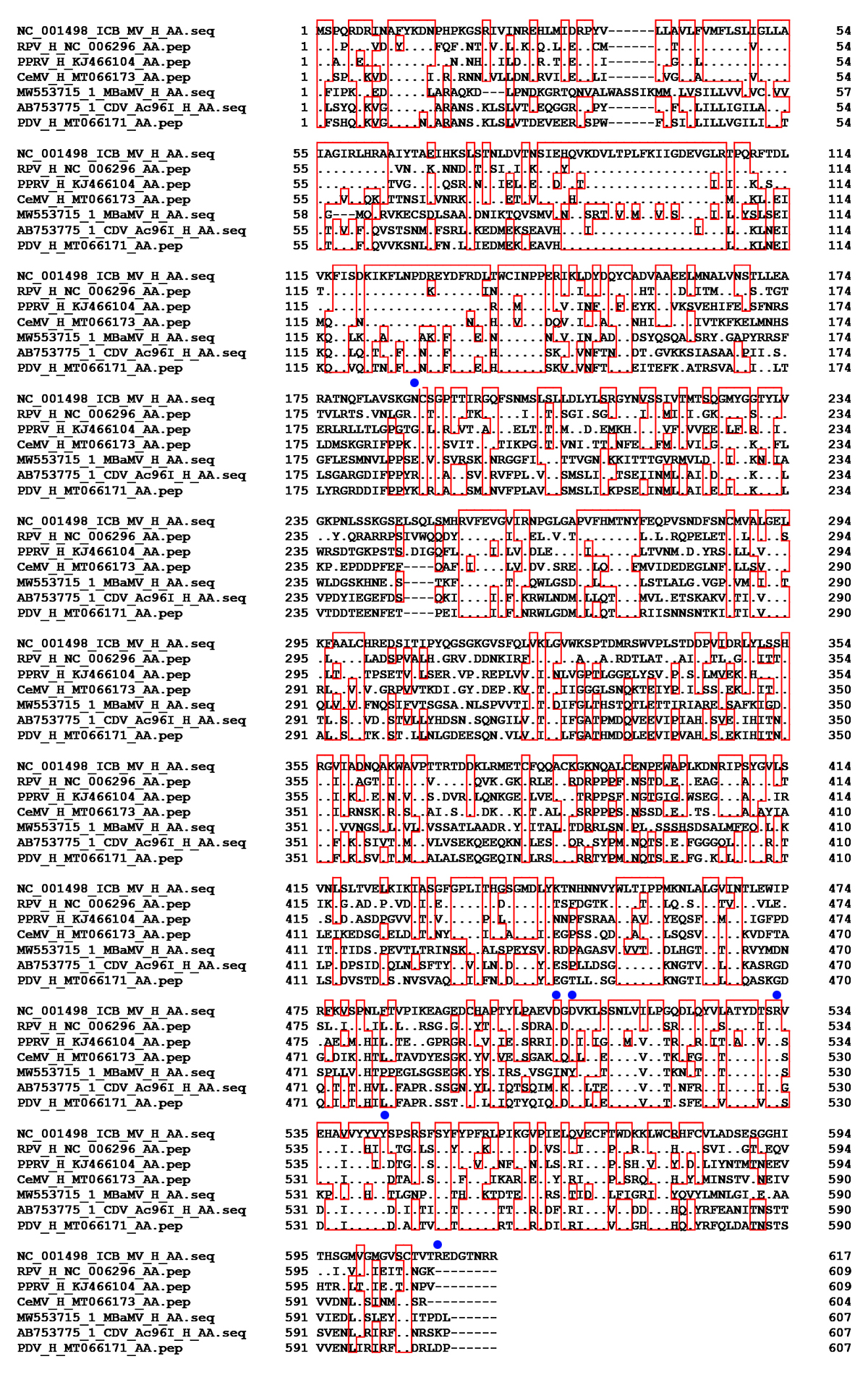

Supplement: S8 Fig — Amino acid sequences of H proteins from seven morbilliviruses were obtained from GenBank and aligned using the ClustalW program in Genetyx-Mac software ver. 21. Residues identical to the top sequence are represented by dots in red boxes, while mismatched residues are shown as their respective amino acid letters. Deleted amino acid residues are indicated by hyphens. The amino acid positions shown to affect the H-SLAM interaction in this study are indicated by blue dots. (TIF) [file ppat.1012990.s010.tif]

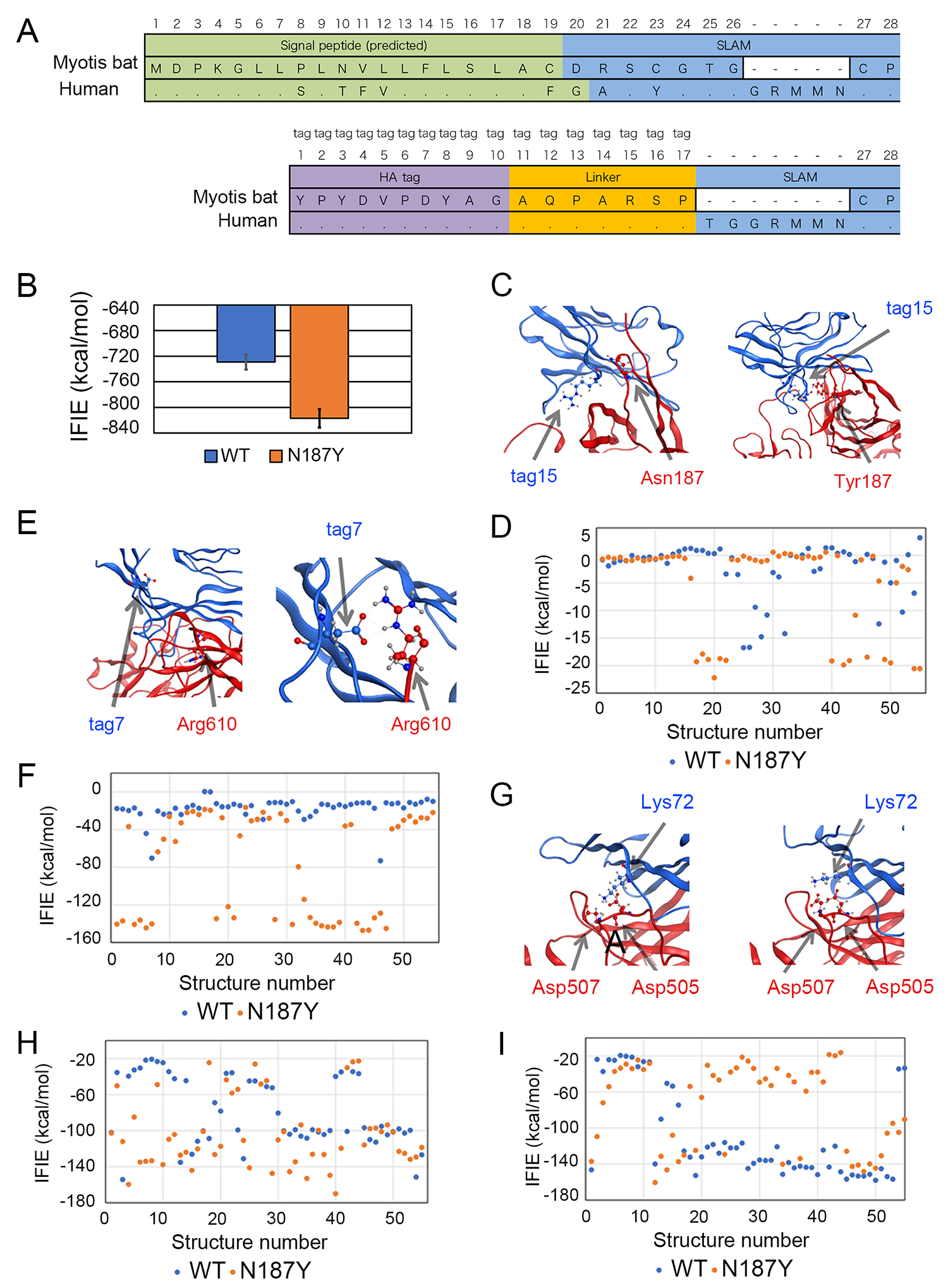

Supplement: S9 Fig — (A) Amino acid sequences of bat and human SLAMs in the absence (upper panel) and presence (lower panel) of a tag and linker. Predicted signal peptide sequence, HA tag sequence, SLAM sequence, and linker connecting the HA tag and SLAM sequence are highlighted in green, purple, blue, and orange, respectively. Residues identical to the top sequence are represented as dots, while mismatched residues are shown as their corresponding amino acid letters. Deleted amino acid residues are indicated by hyphens in the alignment analysis. (B) MD trajectory-averaged total IFIE (kcal/mol) between MV-H and bat SLAM with a tag. Blue and orange bars indicate the FMO-calculated results, showing the mean values and standard deviations, for the wild type (WT) and N187Y mutant, respectively. (C) Positional relationship between H-Asn187 and tag15 (Arg) in bat SLAM with a tag for the wild type (WT) (left) and between H-Tyr187 and tag15 (Arg) for the N187Y mutant (right). (D) Temporal (structure-dependent) variations of IFIE (kcal/mol) between H-Asn187 or H-Tyr187 and tag15 (Arg24) in bat SLAM with a tag. Blue and orange dots represent the wild type (WT) and the N187Y mutant, respectively. The averaged IFIE values are -1.3 kcal/mol for WT and -5.10 kcal/mol for the mutant. (E) Positional relationship between H-Arg610 and tag7 (Asp) in bat SLAM with a tag for WT (left) and for the N187Y mutant (right). (F) Temporal (structure-dependent) variations of IFIE (kcal/mol) between H-Arg610 and tag7 (Asp16) in bat SLAM with a tag. The averaged IFIE values are -18.4 kcal/mol for WT and -78.6 kcal/mol for the mutant. (G) Positional relationship between H-Asp507 or -Asp505 and Lys72 in bat SLAM with a tag for WT (left) and the N187Y mutant (right). (H, I) Temporal (structure-dependent) variations of IFIE (kcal/mol) between H-Asp507 and Lys72 in bat SLAM with a tag (H) and between H-Asp505 and Lys72 (I). The averaged IFIE values are -78.8 kcal/mol and -103.9 kcal/mol for H-Asp507–Lys72 in WT and mutant [file ppat.1012990.s011.tif]

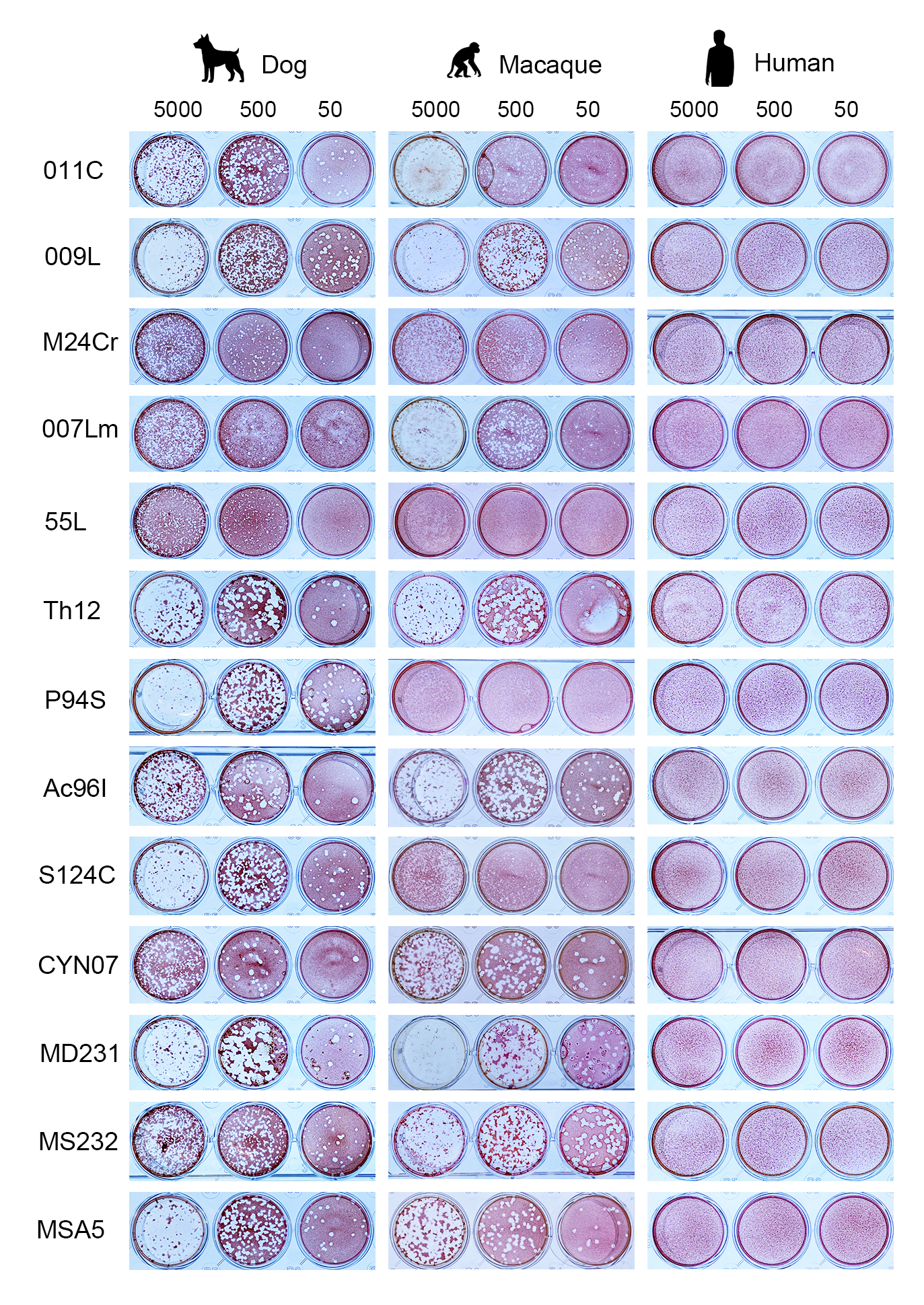

Supplement: S10 Fig — Monolayers of Vero cells stably expressing dog, macaque, and human SLAMs in 12-well cluster plates were infected with different infectious titers (5,000, 500, and 50 plaque-forming units [PFUs]) of each CDV strain and cultured for four days in 1% methylcellulose-containing culture medium. The cells were stained with neutral red to visualize the plaques. Animal silhouette images were generated using OpenAI’s image generation system (DALL·E) and are published under the terms of the Creative Commons Attribution 4.0 International License (CC BY 4.0). For terms of use, see https://openai.com/policies/terms-of-use. (TIF) [file ppat.1012990.s012.tif]

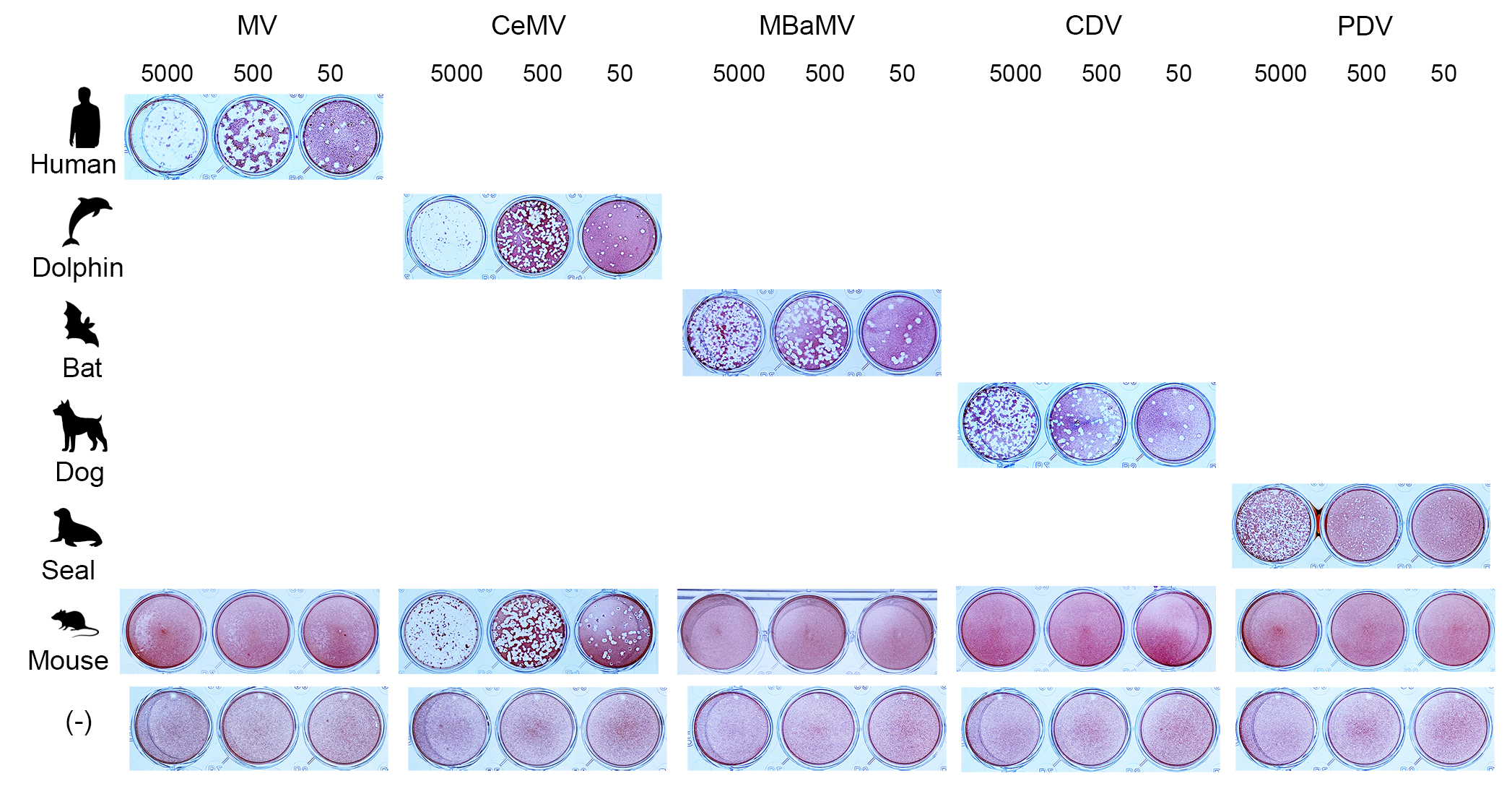

Supplement: S11 Fig — (A) Monolayers of Vero cells stably expressing SLAM from mouse or respective natural host SLAM, along with parental Vero cells (-), were infected with different titers (5000, 500, and 50 plaque-forming units [PFUs]) of each morbillivirus (MV, CeMV, MBaMV, CDV, and PDV) and cultured for four days in culture media containing 1% methylcellulose. Cells were stained with neutral red to visualize plaques. The experiment was triplicated, and representative images are shown. Animal silhouette images were generated using OpenAI’s image generation system (DALL·E) and are published under the terms of the Creative Commons Attribution 4.0 International License (CC BY 4.0). For terms of use, see https://openai.com/policies/terms-of-use. (TIF) [file ppat.1012990.s013.tif]

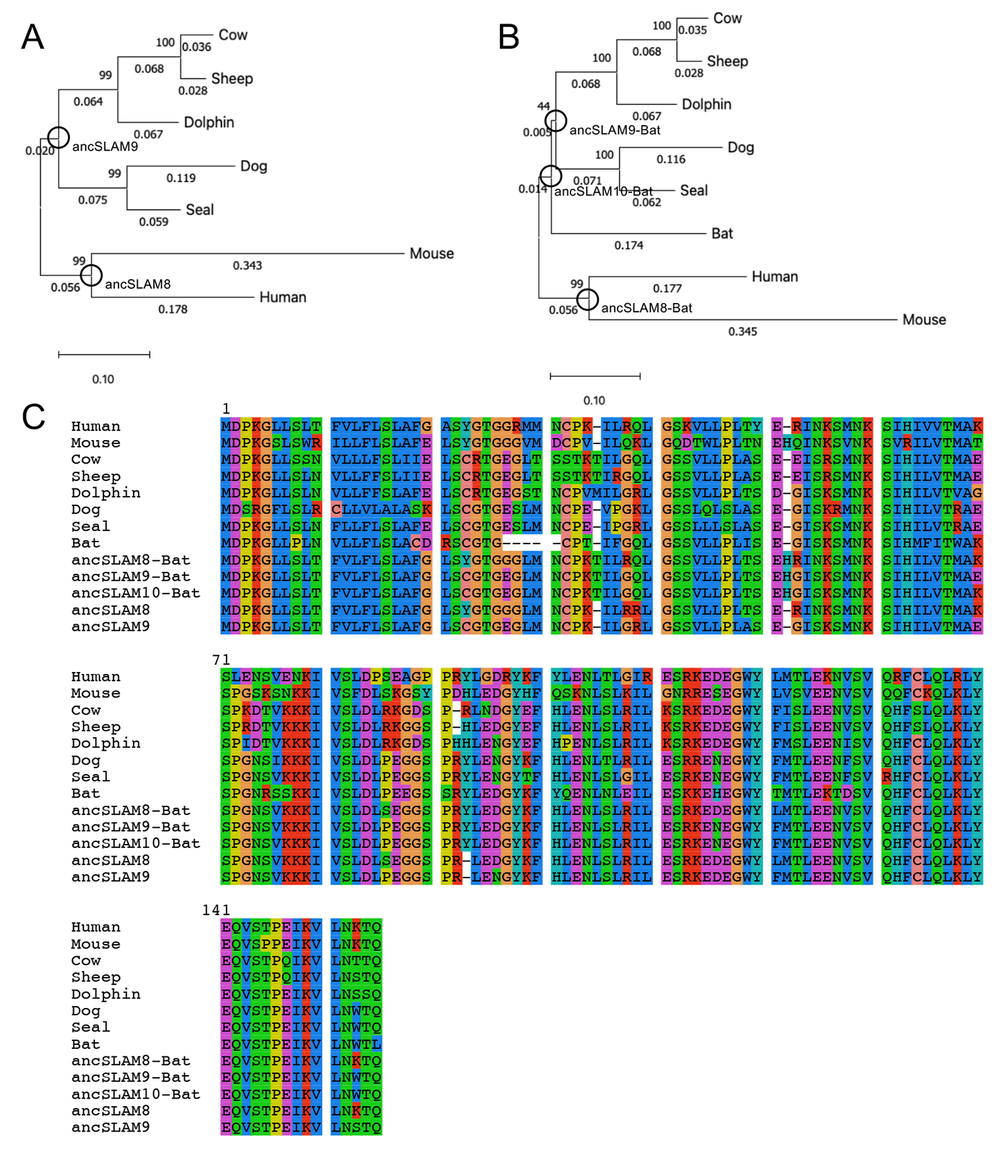

Supplement: S12 Fig — (A) Detailed phylogenetic tree corresponding to Fig 5A. Each branch is labeled with its bootstrap probability based on 1,000 replicates. (B) Phylogenetic tree of animal SLAMs that includes bat, cow, sheep, dolphin, dog, seal, and human. Sequences were aligned with Clustal Omega. The topology was inferred via the neighbor-joining method using the Jones-Taylor-Thornton (JTT) substitution model. (C) Amino acid sequence alignment of the V domains from animal SLAMs (human, mouse, cow, sheep, dolphin, dog, and bat), ancSLAM8, ancSLAM9, and the newly inferred ancestral SLAMs. These ancestral sequences were reconstructed using PAML v4.9j (with the pairwise deletion option) based on an input dataset that included bat SLAM, and were subsequently aligned with Clustal Omega. (TIF) [file ppat.1012990.s014.tif]
