# Supplementary material for: Evolutionary and structural basis of SLAMF1 utilization in morbilliviruses—Implications for host range and cross-species transmission
Source: PLoS Pathog. 2025 Jun 10;21(6):e1012990. doi: 10.1371/journal.ppat.1012990 (PMC12180634; doi:10.1371/journal.ppat.1012990)
Supplement: Attachment — Submitted filename: Response to Reviewers v2.pdf [file ppat.1012990.s017.pdf]

Dear Editor and Reviewers:

We sincerely appreciate your interest in our manuscript and your evaluation that it is worthy of consideration. We carefully considered the constructive and important comments provided by the Editor and the reviewers. All of the requested additional experiments have been performed, and the resulting findings have significantly enhanced the overall quality and impact of the manuscript. We also believe that we have appropriately addressed all the concerns that were raised.

Please note that the line numbers referred to in our "Response to Reviewers" correspond to those in the "Revised Manuscript with Track Changes," not the clean "Manuscript" file.

Best regards

Makoto Takeda

~~~~~~

Reviewers' Comments:

Reviewer #1: In this manuscript, Hyodo et al evaluate whether SLAMs from different species support morbillivirus entry and subsequent replication. Additionally, they perform adaptation experiments of MV to batSLAM, and explore why CDV can use macaqueSLAM but not humanSLAM. The manuscript has interesting findings that are worth publishing, but the data is presented in a rather chaotic form and hard to follow for a general readership.

Answer: We sincerely thank you for recognizing that our study contains findings of sufficient interest to merit publication. We also appreciate your valuable comments regarding the structure of the manuscript. We carefully reviewed and revised the entire manuscript to improve clarity and accessibility, even for readers who may not be specialists in the field. By addressing each of the reviewers' constructive comments in detail, we believe that the flow and significance of our research are now presented more clearly.

Reviewer #2: The authors have studied the interactions of a range of morbilliviruses with CD150 of different host species, using the combination of infection and fusion assays. This study is partially built on previous work of the same authors (Takeda et al., Curr Opin Virol 2020, especially table 1 and figure 2). They identified situations where single point mutations explained if a specific CD150 molecule could be used as cellular receptor. In addition, they showed that in vitro adaptation of viruses to use a novel receptor was straightforward. In addition, the authors constructed hypothetical ancestral CD150 molecules that may have acted as universal morbillivirus receptors.

Answer: We sincerely thank you for your careful and thoughtful review of our manuscript.

Reviewer #3: The study examines the usage of SLAM receptors from different species by measles and the veterinary morbilliviruses. The manuscript is well written and the experiments valid and precisely carried out. However, the evidence of virus growth in the cell lines is primarily based on cell fusion. There is no immunofluorescence data. This is particualry important for PDV, where some wild type strains have been shown to cause cell rounding rather than fusion and to grow to good titres in Vero cells without a SLAM receptor (this has been how in a number of laboratories). The authors need to carry out immunofluorescense staining to confirm their conclusions.
~~~~~~

Answer: We sincerely appreciate your positive evaluation of our study. The points you raised were highly relevant, and we took them seriously. In particular, we performed an additional experiment using immunofluorescence staining to address your specific concern, and the results have been incorporated into the revised manuscript (lines 212–225, Supplementary Fig. 5). We believe that this additional experiment has added further depth to our study and clarified its significance.

Part II – Major Issues: Key Experiments Required for Acceptance

Please use this section to detail the key new experiments or modifications of existing experiments that should be absolutely required to validate study conclusions.

Generally, there should be no more than 3 such required experiments or major modifications for a "Major Revision" recommendation. If more than 3 experiments are necessary to validate the study conclusions, then you are encouraged to recommend "Reject".

Reviewer #1: Major comments
- The authors continuously use different combinations of SLAMs and morbilliviruses, without providing a logical rationale for that. Actually, the authors don't present a clear hypothesis and goal in this manuscript at all. This makes the manuscript chaotic and difficult to read. A clear hypothesis and goal should be added to the abstract and end of introduction.

Answer: We sincerely appreciate your constructive and appropriate comments. We have added our rationale for selecting the specific combinations of SLAM and morbilliviruses in the revised manuscript (lines 178–183 and 445–447). In accordance with your guidance, we have also clearly stated our hypothesis and research objectives at the end of both the Abstract and the Introduction (lines 54–58 and 145–152).

- The authors show that many animal morbilliviruses can use SLAMs from different host species, concluding that morbilliviruses are adaptable. However, human SLAM is inaccessible to any other morbillivirus than MV. So, if the authors want to make a case that animal morbilliviruses could jump to humans (line 84), why was this adaptation to human not investigated? Rather the authors investigated the adaptability of MV to batSLAM and CDV to macaqueSLAM, which seems completely irrelevant? Why was macaque SLAM not included in the first 2/3 figures?

Answer: As the reviewer pointed out, performing adaptation experiments of animal morbilliviruses to human SLAM could have provided a direct assessment of their potential to infect humans, and we agree with this point. However, due to biosafety and ethical concerns related to gain-of-function and dual-use research, we did not conduct such experiments. In particular, given the increasingly strict regulatory oversight of gain-of-function studies involving zoonotic pathogens, we chose to focus our study on naturally occurring variants and adaptation to non-human SLAMs. These considerations have been explicitly stated in the revised manuscript and noted as a limitation of the present study (lines 648–655).

We have added an explanation regarding the rationale for selecting the animal species shown in Figures 1 and 2 in lines 178–183. We chose not to include macaques because the selected species are either known or strongly suspected to be natural hosts of morbilliviruses, and are therefore likely to exhibit high susceptibility to infection. Consequently, prioritizing the evaluation of SLAM receptors from these species provides a biologically and epidemiologically relevant framework for assessing morbillivirus receptor compatibility.

- Why was mouse SLAM suddenly included in the experiments with the ancestral SLAM. Why was it not included in earlier datasets (first 2/3 figures).

Answer: To address the reviewer's concern regarding the sudden inclusion of mouse SLAM in the evaluation of ancestral SLAM receptors, we would like to clarify the conceptual transition in the experimental design. In the earlier phases of this study (Figures 1 and 2), our focus was on evaluating SLAMs from animal species that are known natural hosts of morbilliviruses, thereby emphasizing the ecological and epidemiological relevance of cross-species transmission.

In contrast, the latter part of the study shifts toward an evolutionary framework, aiming to dissect the structural and phylogenetic basis of SLAM function. Within this context, mouse SLAM was included as a representative member of the Rodentia order, which—despite not being a known natural host—provides a valuable comparative reference for reconstructing ancestral SLAM sequences. Specifically, the inclusion of mouse SLAM enabled us to define a divergence point between Primates and Rodentia, which was necessary for the reconstruction of the hypothetical ancestral SLAM8 (ancSLAM8). Therefore, the use of mouse SLAM is justified not by ecological considerations, but by its phylogenetic utility in evolutionary inference.

This shift in experimental emphasis—from host-pathogen interactions in natural settings to the structural evolution of SLAM receptors—explains the apparent discontinuity and highlights the logical coherence of our overall study design.

Accordingly, we have clarified in the manuscript the rationale for selecting specific animal SLAM receptors in Figures 1 and 2 (lines 178–183), as well as the reason for including mouse SLAM at the stage of ancestral SLAM analysis (lines 445–447).

- Splitting the abstract into two pieces, with a second part specifically on bats, does not help readability. The specific focus on the bat results in the abstract, to a detailed level, is unnecessary. The bat data should be integrated, and a single cohesive abstract should be presented.

Answer: We agree that the reviewer's comment is well taken. In accordance with your guidance, we have removed references to bats wherever possible and reframed the related content as an example highlighting the adaptability of morbilliviruses. The Abstract has been revised accordingly.

- Line 118, but also a general remark to the authors findings: the fact that morbilliviruses can often utilize SLAMs from non-native hosts with similar efficiency but are still quite host-restricted, actually suggests that receptor usage might not be the most important host determinant to me. The authors discuss this in lines 386-407, but in a one-sided manner. I would argue that the fact that morbilliviruses rarely establish themselves in a new host species is an argument that these viruses are extremely host-restricted. Can the authors extend this section of the discussion?

Answer: We fully agree with the reviewer's comment. Our findings highlight the intrinsic adaptability of morbilliviruses in utilizing or adapting to SLAM receptors from a wide range of species. However, it is equally important to recognize that successful cross-species transmission events—particularly those leading to sustained transmission within a new host population—remain exceedingly rare. These rare instances suggest that, despite receptor compatibility, morbilliviruses likely face substantial post-entry barriers in non-host species, including species-specific innate immune responses, intracellular restriction factors, and incompatibilities in replication or transmission. Therefore, observed host restriction is likely multifactorial, and receptor usage alone is insufficient to predict zoonotic potential or host-switching events. Nevertheless, adaptation to a new receptor undeniably represents a critical step for a virus to

cross the species barrier. These points have been clearly addressed in the revised manuscript (lines 474–507).

- The data presented in figure 1, 2, and 3 largely show the same results: syncytia in SLAM expressing cells, plaques in SLAM expressing cells, and fusion with SLAM (without HA tag) expressing cells. The authors could condense these data. It is confusing that cow and sheep SLAM were added to figure 3, as they could also have been included in figure 1 and 2.

Answer: In accordance with the reviewer's suggestion, the original Figure 1 has been moved to Supplementary Figure 1 in the revised manuscript. In addition, the original Figures 2 and 3 have been combined into a single figure. The data previously shown in Figures 2B, 2C, and 3B have been removed, and the revised figure is now presented as the new Figure 1.

In the original Figures 1 and 2, infectious virus-based analyses required the establishment of cell lines that stably express SLAM molecules. In this study, we did not generate Vero cell lines stably expressing bovine or ovine SLAM. One major reason for this was the unavailability of infectious viruses such as Rinderpest virus (RPV) and Peste des petits ruminants virus (PPRV), which naturally infect cattle and sheep, respectively. Therefore, to compensate for the lack of analyses using infectious RPV or PPRV in combination with bovine or ovine SLAM—analyses not performed in the original Figures 1, 2, and 3—we employed a plasmid-based transient expression system. These data were presented as Figure 3 in the original manuscript and are now shown as Figure 1B in the revised version.

- What does the fusion assay quantitatively assess? MV seems more fusogenic than other viruses, in conjunction with more efficient plaque formation than other morbilliviruses. What does this mean?

Answer: Since the cell–cell fusion assay using expression plasmids does not depend on viral replication, it enables the evaluation of protein function independently of virus growth—unlike the data shown in the original Figure 1 (observation of CPE) and Figure 2 (plaque formation). This point has been clarified in the revised manuscript (lines 231–234).

- In line 216-231 the authors extensively explain the interaction between MV-H and HAtag-batSLAM. Why is this relevant, and is it not sufficient to present the findings on the interactions between MV-H and untagged batSLAM?

Answer: In the main text, we presented only the interaction data for untagged bat SLAM, while the data for tagged bat SLAM were included in the Supplementary Data.

Reviewer #2: My major criticism to the study is the lack of context of prerequisites for cross-species morbillivirus infection. First, the heterologous target host (or host population) needs to lack morbillivirus-specific immunity. It has been well described that morbillivirus-specific cellular immune responses provide cross-protection to other morbilliviruses. Second, the host needs to be susceptible. I agree with the authors that nectin-4 is much more conserved than CD150, which will likely make CD150-mediated infection of immune cells the critical hurdle. Next, the host cells need to be permissive, and the antiviral proteins need to effectively suppress innate immune responses in the novel host. Finally, and most importantly, the virus needs to be transmitted to the next host. Without onward transmission, all adaptive mutations are lost in the first infected host. All these factors are well-explained in the COV review of 2020 but are lost in the current manuscript.

Answer: We fully agree with the reviewer's comment. Our findings highlight the intrinsic adaptability of morbilliviruses in utilizing or adapting to SLAM receptors from a wide range of species. However, it is equally important to recognize that successful cross-species transmission events—particularly those leading to sustained transmission within a new host population—remain exceedingly rare. These rare instances suggest that, despite receptor compatibility, morbilliviruses likely face substantial post-entry barriers in non-host species, including species-specific innate immune responses, intracellular restriction factors, and incompatibilities in replication or transmission. Therefore, observed host restriction is likely multifactorial, and receptor usage alone is insufficient to predict zoonotic potential or host-switching events. Nevertheless, adaptation to a new receptor undeniably represents a critical step for a virus to cross the species barrier. These points have been clearly addressed in the revised manuscript (lines 474–519). The protective effect conferred by antigenic cross-reactivity is also an important factor, and this point has been addressed in lines 514–516 of the revised manuscript.

The abstract is more a narrative than that it summarizes the work and main findings of this manuscript. As far as I can assess, lines 46-61 does not contain any new information. The adaptation experiment of MV to use myotis bat CD150 if new, but the authors do not explain that this is the inverse of what would be a more interesting experiment (although probably not possible due to concerns about gain of function): how easy is it for the bat virus to adapt to use human CD150? A reverse zoonosis transmission of MV to bats is not a likely event. The hypothetical ancestral CD150 molecules proposed in this manuscript are truly novel, and an original approach tu understanding morbillivirus evolution. However, in my opinion the manuscript whould be rewritten to explain the specific aim, the findings and their novelty, and the advance that the study presents to the existing literature.

Answer: In accordance with the reviewer's suggestion, we have improved the Abstract by removing unnecessary content and refining the wording wherever possible. We also agree that adaptation experiments using human SLAM would provide a more direct approach. In the revised Abstract (lines 63–65), we have clarified that the adaptation of measles virus (MV) to bat SLAM was conducted as an experimental example to show the adaptability of morbilliviruses. In the main text (lines 648–655), we explained that the study design was based on our research objectives and considerations related to gain-of-function experiments. Additionally, we revised the relevant sections (lines 54–57 and 145–152) to clearly state our hypothesis and the corresponding experimental objectives.

Reviewer #3: The authors need to carry out immunofluorescense staining on the infected cell lines to validate their conclusions.

Answer: In particular, we performed an additional experiment using immunofluorescence staining to address your specific concern, and the results have been incorporated into the revised manuscript (lines 212–225, Supplementary Fig. 5). We believe that this additional experiment has added further depth to our study and clarified its significance.

Part III – Minor Issues: Editorial and Data Presentation Modifications
Please use this section for editorial suggestions as well as relatively minor modifications of existing data that would enhance clarity.

Reviewer #1: Minor comments
- English could be improved throughout:
Line 2: 'Its' could be deleted.

Answer: This has been corrected.

Line 48: what does 'severe cross-species transmission' mean?

Answer: The text has been revised as follows: Cross-species transmission events with significant mortality have also been reported.

Line 88: what does 'major pathogen' mean

Answer: The text has been revised as follows: Morbilliviruses, a genus within the Paramyxovirus family, comprise a group of highly pathogenic viruses with significant medical and veterinary relevance.

Line 160 (and other places): rather use 'infectious virus' than 'live virus'
And many other examples

Answer: They have been corrected.

- Line 47: Why did the authors highlight MV, CDV and CeMV

Answer: MV, CDV, and CeMV are listed as examples of morbilliviruses that infect humans, terrestrial animals, and marine mammals, respectively. PPRV is also included, as morbilliviruses infecting livestock are of significant importance

- Line 59-60: 'bat SLAM also functioned as an efficient receptor for multiple morbilliviruses'. Is this background or a finding of this paper? Unclear now.

Answer: The text has been revised as follows: This study showed that bat (Myotis) SLAM also functioned as an efficient receptor for multiple morbilliviruses.

- Line 91: The authors indicate that morbilliviruses lead to economic losses in livestock industries. This only holds true for PPRV, and this should not be a generalized statement.

Answer: The text has been revised as follows: They include pathogens that pose substantial health and economic burdens due to their capacity to cause lethal, widespread outbreaks, leading to high mortality rates and economic losses in livestock industries.

- Line 97: novel morbilliviruses were not discovered in bats or swine, rather the presence of a sequence was demonstrated.

Answer: The text has been revised as follows. The recent discovery of novel morbilliviruses (or their genomic components) in bats (Myotis, Phyllostomus, and Molossus) , swine, and cats suggests a broader host spectrum than previously recognized.

- Line 103: no need to refer to a figure in the introduction.

Answer: This has been corrected.

- Line 113: has CDV jumped to primates since 2010?

Answer: The text has been revised as follows: Notably, CDV caused large-scale outbreaks in non-human primates in 2006–2008

- Line 191: the N187Y mutation does not become directly clear in supplementary figure 5.

Answer: We decided not to cite Supplementary Figure 5 here.

- Line 203-207: no need to extensively repeat the findings of the prior paragraph.

Answer: We have deleted the repeated descriptions of the results.

- The extensive descriptions of IFIEs and PIEDA is rather technical and not relevant for a general readership. Could these results be summarized in a table shortening the results section extensively?

Answer: In accordance with the reviewer's comments, we have extensively revised the manuscript to use plain language that is accessible to readers without expertise in computational science. Additionally, we have organized the data into a table and simplified the description of the results (lines 370–402 and Supplementary Table 1).

- Line 531-533: the phylogenetic tree should be part of the supplemental data.

Answer: Phylogenetic tree data has been added as Supplementary Figure 12A, B.

Reviewer #2:

1. Title: the authors are recommended to use the term CD150. If not, they should use the term SLAMF1.

Answer: We have revised the title by changing "SLAM" to "SLAMF1," and also clarified in the main text (line 53) that Signaling Lymphocytic Activation Molecule (SLAM) is more formally referred to as SLAMF1 or CD150.

2. Line 48: the term "severe" refers to disease, not to transmission events.

Answer: This has been corrected.

3. Lines 81-82: add "in vitro": in vivo cross-species transmission events have been described (and all current morbilliviruses are thought to have evolved after cross-species transmission events and adaptation to novel hosts. However, in vivo these events are still rare, and morbillivirus infections are usually restricted to a well-defined range of host species (as also summarized in the 2020 COV review).

Answer: We agree with the reviewer's comment and have explicitly stated in the manuscript that cross-species transmission of morbilliviruses in nature is a rare event (lines 474–519).

4. Lines 127-146 and Figure 1A: these data are largely identical to Figure 2 in Takeda et al Curr Opin Virol 2020. The only addition is the combination of MBaMV and Vero-batCD150. Figure 1B should be moved to the supplemental data. It mostly shows that the PDV strain used in this study was likely somewhat attenuated.

Answer: Figure 1A in the original manuscript has been moved to Supplementary Figure 4 in the revised version. We have also added a statement in the main text (lines 212–214) noting that the cytopathic effect of PDV used in this study was weaker than that of other morbilliviruses.

5. Lines 147 and Figure 2: it is unclear to me what the added value is of the plaque assay over the syncytium assay shown in Figure 1. It is unclear why MV did not infect Vero-batCD150 in the first study but still caused small plaques in the plaque assay. This is not explained by the authors.

Answer: In bat SLAM cells, small syncytia and cell rounding were occasionally observed in certain areas; however, the cytopathic effect (CPE) was extremely limited, and no apparent CPE was detected in most microscopic fields. This point has been explicitly stated in the revised manuscript (lines 193–195).

While we agree that observations of CPE and plaque formation reflect closely related phenomena, there are cases in which viruses that induce only minimal CPE still form relatively

large plaques, highlighting distinct characteristics between the two assays. Moreover, CPE observation does not allow for quantitative evaluation, whereas plaque assays enable the quantification of infectious virus particles and the extent of infection spread. Therefore, we believe that presenting both types of data is meaningful and justified.

6. Lines 174-178: have the authors considered testing alternative H and F sequences in the DSP assay?

Answer: We also wished to use an RPV sequence that could be confirmed as truly wild-type; however, this was not feasible. The only available option for us was to use the Kabete O strain, which is considered to retain wild-type characteristics. We were unable to identify any other wild-type sequences that were available.

7. Lines 179-181: the rationale of this adaptation experiment is not well-explained. The results of the experiment are convincing, and well-presented.

Answer: We have clearly stated in the manuscript that this experiment was conducted as an example demonstrating the ability of morbilliviruses to readily adapt to SLAM receptors from different host species (lines 63–64 and 276–278).

Indeed, experimental adaptation of animal-derived morbilliviruses to human SLAM or human cells would allow a more direct assessment of their potential for zoonotic transmission. However, such studies raise significant biosafety and ethical concerns, particularly in the context of gain-of-function (GoF) research and dual-use research of concern (DURC), and therefore were not performed in this study.

Given the increasing regulatory oversight of GoF experiments involving potential zoonotic pathogens, we instead focused on naturally occurring variants and their adaptation to non-human SLAM receptors. This strategy enabled us to investigate receptor usage and host range within a framework that balances scientific rigor with ethical responsibility. We have stated this in the manuscript (lines 648–655).

8. Lines 203-241: the computational analysis presented in Fig 5 is convincing, but the importance is not well-explained.

Answer: The significance of computational analysis has been clearly stated (lines 324–331). The statement is as follows: Many previous studies have shown that morbilliviruses can change the ability to utilize SLAM through a single amino acid substitution in the H protein. However, detailed molecular analyses of how these point mutations alter the interactions between the H protein and SLAM at the molecular level remain limited. In this study, we present one example, clearly demonstrating that a single amino acid change can influence multiple intermolecular interactions. Importantly, this study underscores the potential of computational approaches in elucidating the mechanisms of viral adaptation to new host receptors (lines 324–331).

9. Lines 303-308: the authors observed "reduced receptor functionality" in cells expressing macaque slam wit Arg28 but conclude that "human SLAM cannot serve as a CDV receptor due to the presence of R28". The reduced receptor functionality in Fig 6E is substantially higher than the negative control, how can the authors exclude that the virus can adapt? In fact, this has previously been described in references 44 and 45 (see also discussion, lines 383-385).

Answer: We fully agree with the reviewer's comment. Accordingly, we have added the following statement to the revised manuscript: However, it should be noted that although

receptor functionality was reduced, a clear distinction remained when compared to SLAM-negative cells, indicating that the receptor was not wholly non-functional.

10. Lines 324-326: the exclusion of bat CD150 in this analysis is unfortunate, as bats are considered potential hosts of ancestral morbilliviruses (see also lines 357-358). This point should be addressed in the discussion.

Answer: We agree with the reviewer's comments, but for the following reasons, we have excluded bat SLAM from our ancestral SLAM prediction analysis. We have also clearly stated these reasons in the revised text (lines 454–460).

First, bat SLAM has a five-amino-acid deletion at its N terminus (Supplementary Fig. 1), and PAML does not have models that accommodate insertions or deletions (1). Because an alignment gap is treated as an ambiguity (i.e., five question marks "?"), the N-terminal deletion in bat SLAM is simply represented by those consecutive gaps. When PAML handles ambiguities, one can choose pairwise deletion (removing only sites with missing characters in each pair) or complete deletion (removing all sites with ambiguity characters and alignment gaps across every sequence). However, neither approach is an evolutionarily meaningful model. In the PAML output, the phrase "Unreliable at sites with alignment gaps" is explicitly noted. Given that the N-terminal region, which is missing in bat SLAM, plays an essential role in determining receptor usage for CDV (Fig. 4E), we concluded that including bat SLAM for ancestral SLAM reconstruction would likely affect the results.

Second, the placement of bat SLAM in the phylogenetic tree is controversial. Since we confirmed that SLAM evolution follows additivity, the neighbor-joining (NJ) method can reconstruct the tree accurately (2). Nevertheless, when bat SLAM was included, the bootstrap value for the branch uniting cow, sheep, dolphin, dog, and sheep SLAMs dropped (Supplementary Fig. 12B). This decrease did not occur in the tree without bat SLAM, suggesting that bat SLAM complicates topology determination. As nodes with low bootstrap support have limited interpretive value, we conclude excluding bat SLAM gives more robust results overall.

1) Yang Z. PAML: Phylogenetic Analysis by Maximum Likelihood. Version 4.9j (February 2020) [Internet]. 2020 [cited 2025 Apr 6]. p.12. Available from: http://abacus.gene.ucl.ac.uk/software/pamlDOC.pdf

2) Studier JA, Keppler KJ. A note on the neighbor-joining algorithm of Saitou and Nei. Mol Biol Evol. 1988 Jul 1;5(6):729–31.

11. Lines 348-352: CDV is considered as a more recently diverged morbillivirus than MV (Uhl et al 2019, PMID: 30743216). Does this fit the observed picture?

Answer: We have cited the paper by Uhl et al. and also referred to it in the main text (lines 511–512).

12. Lines 386-397: this paragraph should address cross-protective immunity.

Answer: We agree with the reviewer's comments, and we did mention the antigenic cross-reactivity in the revised main text (lines 514–516).

13. Lines 398-399: only if combined with transmissibility to the next host, see comment 1. As transmission happens relatively late in the viral life cycle, there are several other restrictions. Receptor adaptation is necessary but not sufficient.

Answer: We fully agree with the reviewer's comment. Our findings highlight the intrinsic adaptability of morbilliviruses in utilizing or adapting to SLAM receptors from a wide range of species. However, it is equally important to recognize that successful cross-species transmission events—particularly those leading to sustained transmission within a new host population—remain exceedingly rare. These rare instances suggest that, despite receptor compatibility, morbilliviruses likely face substantial post-entry barriers in non-host species, including species-specific innate immune responses, intracellular restriction factors, and incompatibilities in replication or transmission. Therefore, observed host restriction is likely multifactorial, and receptor usage alone is insufficient to predict zoonotic potential or host-switching events. Nevertheless, adaptation to a new receptor undeniably represents a critical step for a virus to cross the species barrier. These points have been clearly addressed in the revised manuscript (lines 474–507).

14. Lines 408-411: I agree with the statement but disagree with the conclusion that this study provides essential scientific evidence for the importance of CD150-adaptation as the basis of cross-species transmission.

Answer: We agree with the reviewer's opinion. We have rewritten the conclusion as follows: In conclusion, this study shows that evolutionarily conserved structural features primarily govern SLAM's receptor function for morbilliviruses. These features form the basis for the cross-species transmission potential of morbilliviruses. While receptor usage constitutes only one of several barriers to cross-species transmission, this study uncovers SLAM's structural and functional properties and the basis of its adaptability as a morbillivirus receptor. These data are critically important for assessing the zoonotic potential of morbilliviruses and for understanding past and future events of cross-species transmission of these viruses.
