# Supplementary material for: Evolutionary and structural basis of SLAMF1 utilization in morbilliviruses—Implications for host range and cross-species transmission
Source: PLoS Pathog. 2025 Jun 10;21(6):e1012990. doi: 10.1371/journal.ppat.1012990 (PMC12180634; doi:10.1371/journal.ppat.1012990)
Supplement: Attachment — Submitted filename: Response to Reviewers (2nd revise).pdf [file ppat.1012990.s018.pdf]

Dear Editor and Reviewers:

We sincerely appreciate your evaluation that our work is worthy of consideration. We have carefully reviewed the constructive and important comments provided by the Editor and the reviewers, and have revised the manuscript accordingly. These modifications have improved the overall quality and impact of the manuscript. We believe that we have thoroughly addressed all the concerns that were raised.

**Please note that the line numbers referred to in our "Response to Reviewers" correspond to those in the "Revised Manuscript with Track Changes," not the clean "Manuscript" file.**

Best regards

Makoto Takeda

~~~~~~~~~~~~~~~~~~~~~~~~~~~~~~~~~~~~~~~~~~~~~~~~~~~~~~~~~~~~~~~~~

**Reviewers' Comments:**

**Reviewer #1: In this manuscript, Hyodo et al evaluate whether SLAMs from different species support morbillivirus entry and subsequent replication. Additionally, they perform adaptation experiments of MV to batSLAM, and explore why CDV can use macaqueSLAM but not humanSLAM. The manuscript has interesting findings that are worth publishing, but the data is presented in a rather chaotic form and hard to follow for a general readership.**

Answer: We sincerely thank you for recognizing that our study contains findings of sufficient interest to merit publication. We also appreciate your valuable comments regarding the structure of the manuscript. We carefully reviewed and revised the entire manuscript to improve clarity and accessibility, even for readers who may not be specialists in the field. By addressing each of the reviewers' constructive comments in detail, we believe that the flow and significance of our research are now presented more clearly.

**Reviewers' Comments:**

**Part I – Summary**

**Reviewer #3: The authors have made major revisions to the manuscript, including adding the requested immunofluorescence experiments. The manuscript is therefore greatly improved. However, a remaining point that needs addressed is that on page 8 relating to supplementary figure 5, there is some infection at 18 hours in the parental Vero cells, indicating that another receptor rather than SLAM may be used. In fact, it has been shown in other studies that if you leave the infection longer it will spread throughout the monolayer and give good virus titres. Therefore while SLAM usage certainly gives better fusion and faster virus spread SLAM may not be necessary for infection of these viruses. This point needs addressed.**

Thank you very much for the reviewer's valuable suggestion regarding the infection evaluation experiment using immunofluorescent staining. The comment has helped us improve the quality of the manuscript. As the reviewer correctly pointed out, a certain level of infectivity was observed even in parental Vero cells in this experiment (Supplementary Figure 5). This indicates that SLAM expression is not an absolute requirement for infection in Vero cells. Rather, SLAM functions as a factor that significantly enhances infection efficiency. To clarify this point, we have added the following sentence to the main text (lines 184–188):
~~~~~~~~~~~~~~~~~~~~~~~~~~~~~~~~~~~~~~~~~~~~~~~~~~~~~~~~~~~~~~~~~

“Indeed, for MV, CeMV, and PDV, infection was consistently observed in Vero cells lacking SLAM expression, at approximately 3–25% of the efficiency in cells expressing the corresponding host animal’s SLAM. These findings suggest that while SLAM is essential for cell-to-cell fusion, it is not strictly required for viral entry, but rather serves as a factor that markedly enhances infection efficiency.”

**Part III – Minor Issues: Editorial and Data Presentation Modifications**

**Reviewer #1:**

**• The abstract has improved majorly, but I find it quite lengthy. I suggest the authors condense line 58-89 to highlight their most important novel findings.**

Thank you again for the reviewer’s insightful comment. We agree with the point. Following the suggestion, we revised the section to remove less essential parts and aimed to make the description more concise (lines 56–63).

**• The authors call the morbillivirus evolution a ‘recent example’ (line 510-511). I guess time is relative, but calling 600 BC recent might not be appropriate.**

We also agree with the reviewer’s comment regarding the use of the word "recent." We have removed the term from line 381.

**• Line 518-519: I find it not strong to end this section with a question, and suggest the authors revise that sentence accordingly.**

Lastly, we agree with the reviewer’s suggestion for the revision at lines 391–392 and have modified the text accordingly.
